# Supplementary material for: Microstructural constraints on magmatic mushes under Kīlauea Volcano, Hawaiʻi
Source: Nat Commun. 2020 Jan 7;11:14. doi: 10.1038/s41467-019-13635-y (PMC6946699; doi:10.1038/s41467-019-13635-y)
Supplement: Supplementary file 2 — Supplementary Information [file 41467_2019_13635_MOESM2_ESM.pdf]

Supplementary Information: Microstructural Constraints on Magmatic Mushes under Kīlauea Volcano, Hawaiʻi

Penny E. Wieser[*1], Marie Edmonds[1], John Maclennan[1] and John Wheeler[2]

[1] Department of Earth Sciences, University of Cambridge, Downing Street, Cambridge, UK, CB2 3EQ.

[2] Department of Earth, Ocean and Ecological Sciences, University of Liverpool.

* Corresponding author: pew26@cam.ac.uk

Supplementary Tables

Supplementary Table 1 – Spatial and temporal information for the samples used in this study.

Sample	Eruption Date	Description	Location	GPS co-ordinates
KL0909	May 24th, 1969	Ep. 1 Mauna Ulu	ERZ	19˚ 21.826‘ N, 155˚ 12.877‘ W
KL0908	Dec 30th, 1969	Ep. 12 Mauna Ulu	ERZ	19˚ 20.839‘ N, 155˚ 12.518‘ W
KL0910	Nov 10th, 1973	Pau'ahi Crater	ERZ	19˚ 22.313‘ N, 155˚ 13.510‘ W
KL0914	Sept 24-29th, 1971		Intracaldera	19˚ 24.580‘ N, 155˚ 16.631‘ W
KL0916	Aug 14th, 1971		Intracaldera	19˚ 24.137‘ N, 155˚ 16.644‘ W
KL0917	Late July, 1974		Intracaldera	19˚ 24.06‘ N, 155˚ 16.653‘ W
KL0919	Late Dec, 1974	Ka'u Desert	SSWRZ	19˚ 22.649‘ N, 155˚ 17.609‘ W
KL0920	Late Dec, 1974	Ka'u Desert	SSWRZ	19˚ 22.603‘ N, 155˚ 17.713‘ W
KL0921	~1700 AD	Circumferential flow, Ka'u Desert	SSWRZ	19˚ 22.989‘ N, 155˚ 17.464 ‘W
KL0922	Sept 1974		Intracaldera	19˚ 24.201' N, 155˚ 17.502' W
KL0924	Late July, 1974		Extracaldera	19˚ 24.142' N, 155˚ 16.896' W
KL0930	1919-1920	Mauna Iki	NSWRZ	19˚ 21.230' N, 155˚ 23.892' W
KL0931	Sept 24-29th, 1971		NSWRZ	19˚ 20.625' N, 155˚ 21.659' W

Supplementary Table 2 – EBSD acquisition parameters.

SEM Settings		EBSD Settings	
Aperture	*3*	Sample-Detector Distance	*~14 mm*
Voltage	*20 kV*	Background (frames)	*2*
Beam Size	*5.5*	Gain	*10-30*
Sample Tilt	*70˚*	Hough Resolution	*60*
Working Distance	*~17-14 mm*	Band Detection (min/max)	*Min 6 max 12*
Step size (um)	*3-15 µm*	Reference	Forsterite (Fe 0.2 MgO 1.8) AMSDB-ID: 0008912
		EBSP resolution	*320x240 pixels*

Supplementary Table 3 – Calibration materials, count times, and estimates of analytical precision and accuracy for EPMA analysis of olivine crystals. Precision and accuracy was estimated from repeated measurements of a San Carlos Olivine secondary standard. Some instrument drift occurred in session 2. We only report forsterite contents from this session (forsterite contents measured in repeated analyses of San Carlos olivine were used to create a linear interpolation model to correct instrument drift). The drift was less coherent for Si and Ca, so these elements are not reported. Ca contents for run 1 and 3 were internally corrected to the values measured in the San Carlos olivine standard (factors of 1.3 and 1.05 respectively).

**Element**	**Calibration**	**Crystal**	**Peak Count time**	**Analysis order**	**Condition**	**Precision (%) (Std dev/ mean)**	**Accuracy (%) (measured / standard)**
Session 1 – 30nA, 15kV. November, 2017							
Si	Diopside	TAP	10s	1st	N/A	0.49	99.0
Mg	St. Johns Olivine	TAP	20s	2nd	N/A	1.16	97.7
Fe	Fayalite	LIF	20s	1st	N/A	1.07	99.3
Ca	Diopside	LPET	60s	1st	N/A	9.3	130.7
Session 1 – February 2018 – Dual condition run<br>Cond1 = 30nA, 15kV; Cond 2 = 100nA, 15kV							
Si	Diopside	TAP	10s	1st	1	1.12	99.26
Mg	St. Johns Olivine	TAP	20s	2nd	1	2.41	100.30
Fe	Fayalite	LIF	20s	1st	1	1.14	98.47
Ca	Diopside	LPET	10s	1st	2	12.3	118.0
Session 3 –40nA, 15kV. March 2018,							
Si	Diopside	TAP	20s	1st	N/A	0.67	100.7
Mg	St. Johns Olivine	LTAP	20s	1st	N/A	0.72	99.9
Fe	Fayalite	LIF	20s	1st	N/A	0.93	100.58
Ca	Diopside	PET, LPET	30s, 20s	1st	N/A	0.93	105.4

Supplementary Table 4 - Calibration materials and count times for EPMA analyses of matrix glasses.

**Element**	**Calibration Material**	**Crystal**	**Peak Count Time**	**Analysis order**
Na	Jadeite	LTAP	10s	1st
Al	Corundum	LTAP	20s	2nd
Ca	Diopside	PET	20s	1st
P	Apatite	PET	60s	2nd
K	K feldspar	LPET	10s	1st
Ti	Rutile	LPET	60s	2nd
Si	Diopside	TAP	10s	1st
Mg	St. Johns Olivine	TAP	20s	2nd

Fe	Fayalite	LIF	20s	1st
Cr	Cr spinel	LIF	40s	2nd
Mn	Manganese	LIF	40s	3rd

Supplementary Table 5 - Precision and accuracy for EPMA analyses of matrix glasses and melt inclusions. Precision and accuracy was estimated from repeated measurements of VG2 and Basalt_113716-1 secondary standards.

Element	SiO_2	MgO	Al_2O_3	CaO	TiO_2	FeO	Cr_2O_3	MnO	Na_2O	K_2O	P_2O_5
% Accuracy BCR	98.3	98.3	101.5	99.2	101.2	100.7	279.5	85.1	101.9	99.8	84.9
% Precision BCR	1.5	1.6	1.1	1.7	2.3	2.7	115.8	15.8	6.4	11.6	20.6
% Accuracy Indian Ocean Basalt_113716-1	99.2	98.5	101.7	99.0	103.6	101.3	108.8	125.8	100.5	91.0	104.3
% Precision Indian Ocean Basalt_113716-1	1.4	2.2	1.7	1.8	1.8	2.8	66.2	15.4	2.2	17.1	23.4

Supplementary Table 6-Details of spectrometers used for WDS EPMA maps.

**Element**	**Spectrometer**
Al	LTAP
Ca	PET
P	LPET
Al	TAP
Fe	LIF

Supplementary Figures

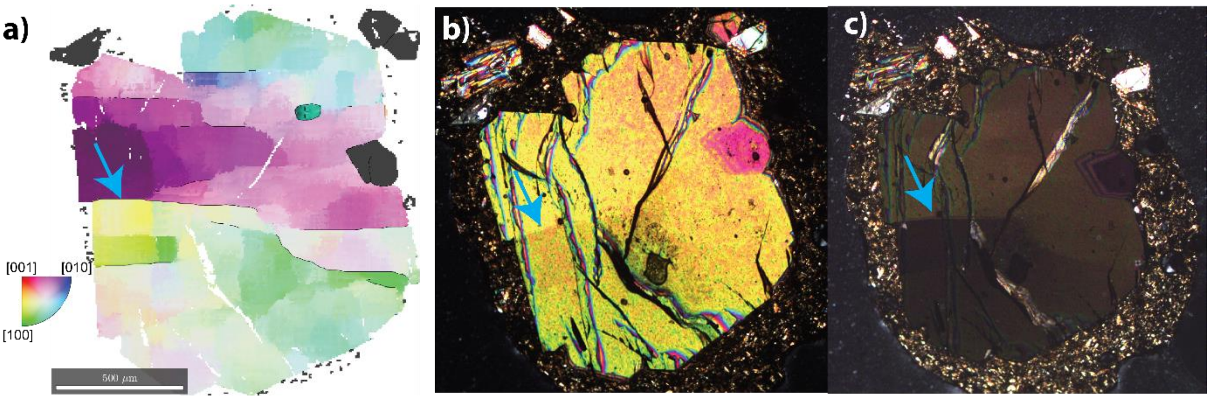

Supplementary Figure 1– Comparison of EBSD-derived IPF map (panel **a**; scaled to 2˚) with photos taken using a high-spec optical microscope (panels **b-c**; Zeiss Axioscope A1). Only a small number of the prominent lattice distortions observed in panel **a)** are visible when viewed with an optical microscope (and only within 5˚ of the extinction position. Additionally, those visible in **c)** when the crystal is close to extinction can only be seen when the brightness is turned all the way up. This shows that unless each grain in a thin section is examined individually (and rotated through 90˚ while constantly adjusting the brightness and contrast), it is exceptionally easy to miss the presence of intracrystalline distortions. Even when care is taken, only distortions with large misorientations (e.g. purple to yellow colors in **a**) are observed.

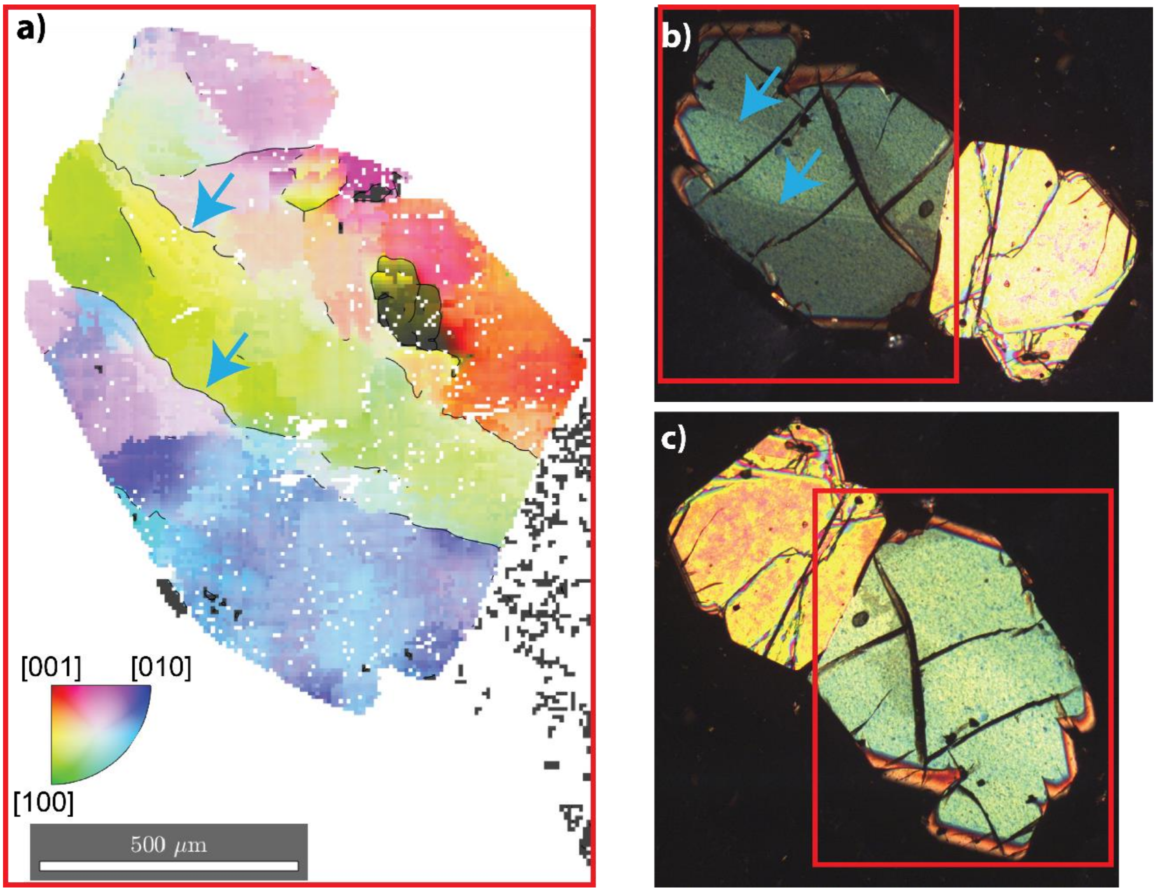

Supplementary Figure 2— Comparison of EBSD-derived IPF map (panel **a**; scaled to 2˚) with photos taken using a high-spec optical microscope (panels **b-c**; Zeiss Axioscope A1). The prominent lattice distortions observed in a) are almost invisible when viewed with an optical microscope. The two most prominent extinction discontinuities (blue arrows) are only visible when the crystal is within 4˚ of its extinction position **(b)**. In fact, unless the brightness of the microscope is adjusted (as is it in image b), it is almost impossible to see these faint color differences.

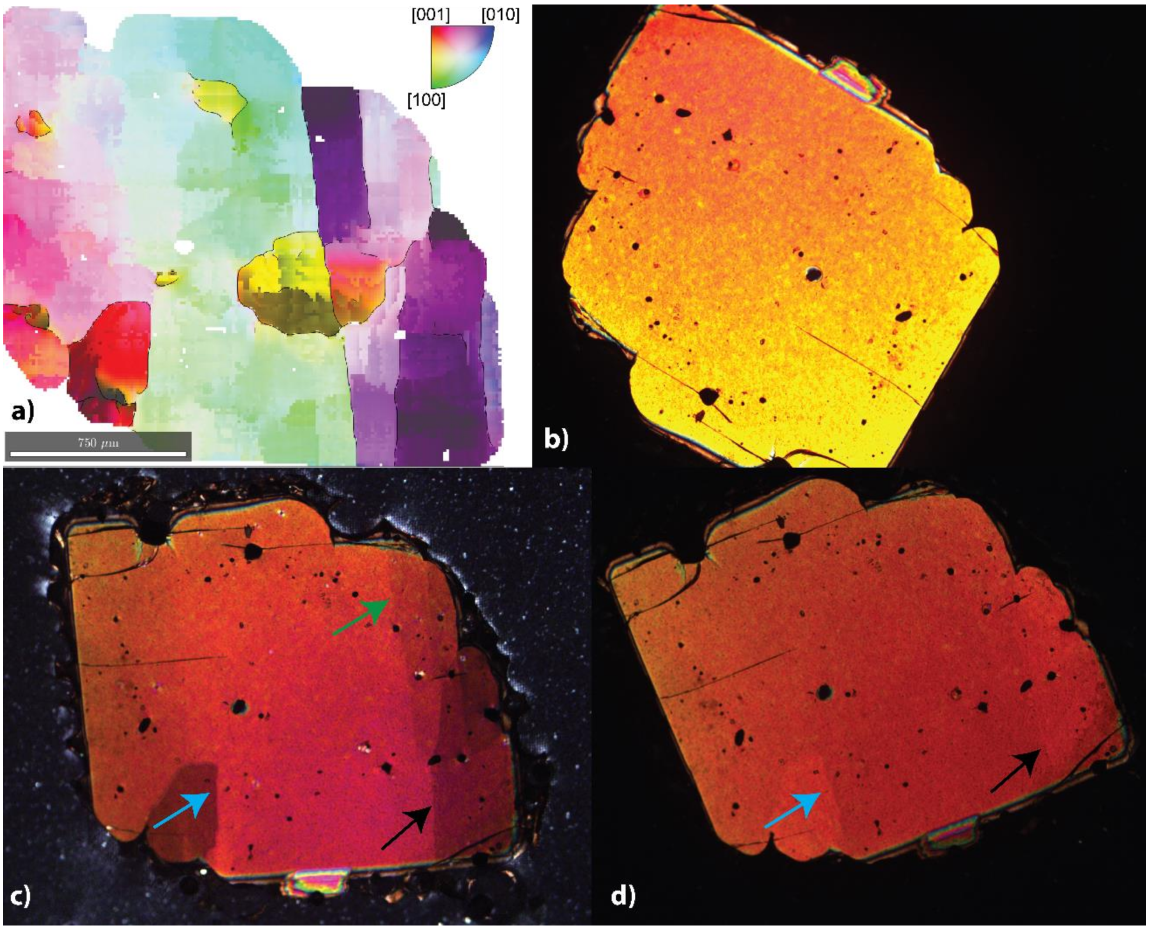

Supplementary Figure 3– Comparison of EBSD-derived IPF map (*panel* **a**; scaled to 2˚) with photos taken using a high-spec optical microscope (*panels* ***b-d***; Zeiss Axioscope A1). The vast majority of low angle boundaries are almost invisible optically (including the prominent transition between green and purple colors in **a**). A few are just visible within 5 ˚ of the extinction position **(c)**, but only when the brightness of the microscope is turned up.

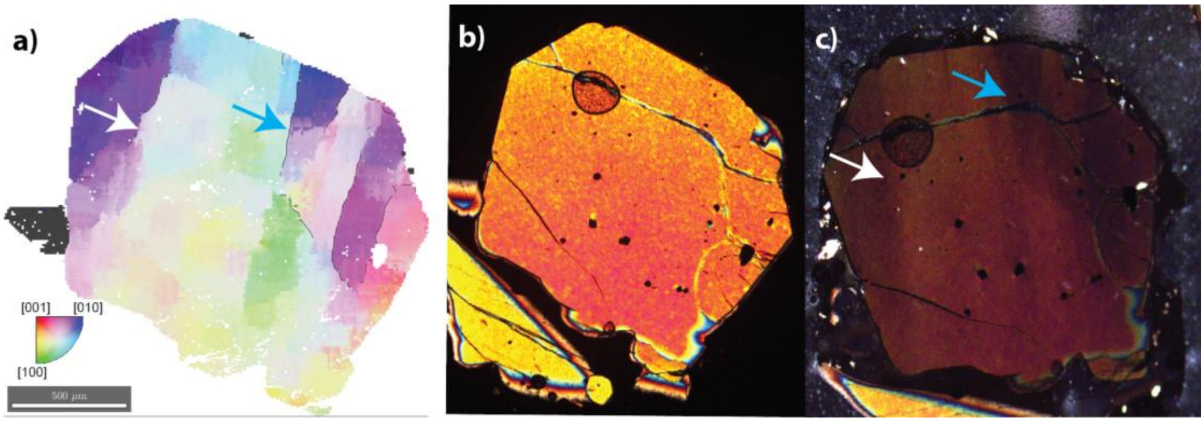

Supplementary Figure 4– Comparison of EBSD-derived IPF map (*panel* **a**; scaled to 2˚) with photos taken using a high-spec optical microscope (*panels* ***b-c***; Zeiss Axioscope A1). Low angle boundaries are visible within 4˚ of the extinction position **(c)**, but only when the brightness of the microscope is turned up to the max, and the brightness is increased dramatically in the imaging software.

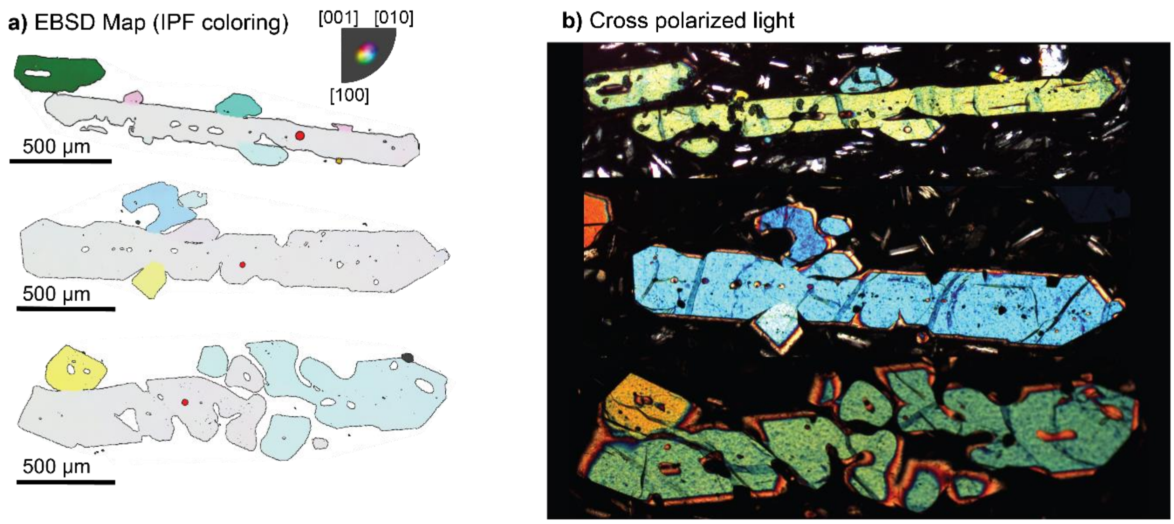

Supplementary Figure 5– Comparison of EBSD-derived IPF map (*panel* ***a****;* scaled to 20˚) with photos taken using a high-spec optical microscope (*panels* ***b-c****;* Zeiss Axioscope A1). Optical photographs of dendritic olivines shown in Fig. 3. The subtle differences in orientation between dendritic branches are difficult to spot (e.g. light blue and white colors in between the left hand and right hand parts of the bottom dendrite).

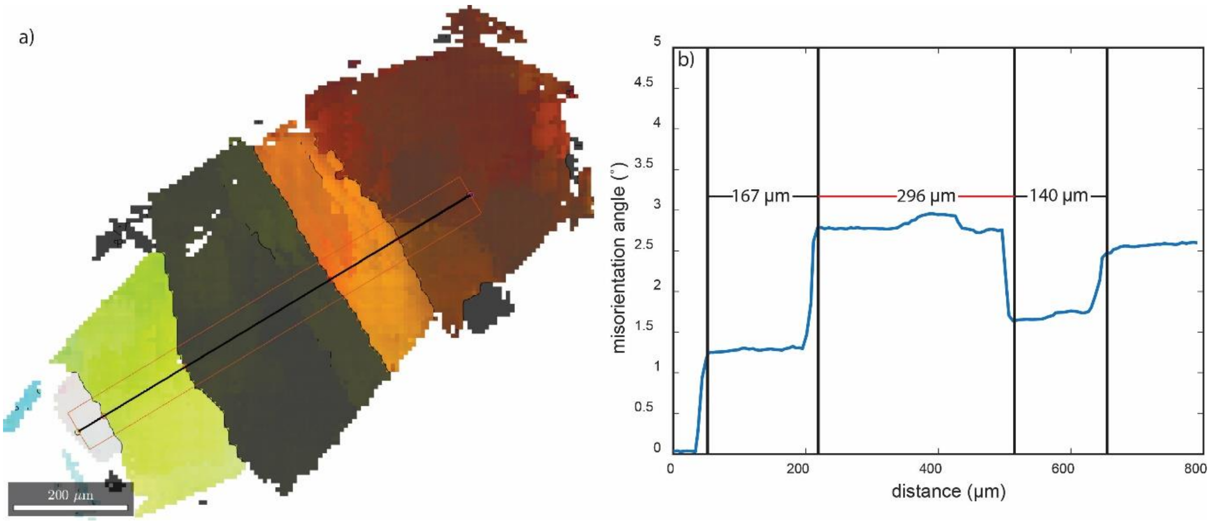

Supplementary Figure 6– Example linear intercepts for one grain. **a)** The black line on the IPF-colored EBSD map denotes the user selected profile, chosen to be nearly perpendicular to subgrain boundaries. **b)** Misorientations along the profile, averaged laterally over 5 pixels either side of the boundary to remove noise (code adapted from https://gist.github.com/kilir/410bab7e3e2777200c5e509f1906d225.). The black lines mark the point at which the low angle boundary is crossed at the centre of the profile.
