# Supplementary material for: Microstructural constraints on magmatic mushes under Kīlauea Volcano, Hawaiʻi
Source: Nat Commun. 2020 Jan 7;11:14. doi: 10.1038/s41467-019-13635-y (PMC6946699; doi:10.1038/s41467-019-13635-y)
Supplement: Supplementary file 3 — Description of Additional Supplementary Files [file 41467_2019_13635_MOESM3_ESM.pdf]

Description of Additional Supplementary Files

File Name: Supplementary Data 1
Description: Olivine and glass compositions are presented as an excel spreadsheet.

File Name: Supplementary Data 2
Description: An example ctf EBSD file for use with the slip system identification code.

File Name: Supplementary Data 3
Description: The slip system identification code provided as a matlab script.
